# Tianma Gouteng Decoction Exerts Pregnancy-Protective Effects Against Preeclampsia *via* Regulation of Oxidative Stress and NO Signaling

**DOI:** 10.3389/fphar.2022.849074

**Published:** 2022-03-21

**Authors:** Meiyuan Jin, Bin Cao, Chao Lin, Jiayong Li, Qiang Xu, Qianlei Ren, Shouying Xu, Chao Tang

**Affiliations:** ^1^ National Clinical Research Center for Child Health of the Children’s Hospital, Zhejiang University School of Medicine, Hangzhou, China; ^2^ Department of Obstetrics, Tongde Hospital of Zhejiang Province, Hangzhou, China; ^3^ Zhejiang Provincial Center for Disease Control and Prevention, Hangzhou, China; ^4^ Department of Neurosurgery, The Children’s Hospital, Zhejiang University School of Medicine, Hangzhou, China; ^5^ Department of Ophthalmology, Hangzhou Traditional Chinese Medicine Hospital Affiliated to Zhejiang Chinese Medical University, Hangzhou, China

**Keywords:** preeclampsia, ROS, NO, tianma gouteng decoction, trophoblast

## Abstract

Preeclampsia (PE), a pregnancy-specific syndrome with the major molecular determinants of placenta-borne oxidative stress and consequently impaired nitric oxide (NO) generation, has been considered to be one of the leading causes of maternal morbidity as well as mortality and preterm delivery worldwide. Several medical conditions have been found to be associated with increased PE risk, however, the treatment of PE remains unclear. Here, we report that Tianma Gouteng Decoction (TGD), which is used clinically for hypertension treatment, regulates oxidative stress and NO production in human extravillous trophoblast-derived TEV-1 cells. In human preeclamptic placental explants, reactive oxygen species (ROS) levels were elevated and NO production was inhibited, while TGD treatment at different periods effectively down-regulated the H_2_O_2_-induced ROS levels and significantly up-regulated the H_2_O_2_-suppressed NO production in human TEV-1 cells. Mechanistically, TGD enhanced the activity of total nitric oxide synthase (TNOS), which catalyze L-arginine oxidation into NO, and simultaneously, TGD promoted the expression of neuronal nitric oxide synthase (nNOS) and endothelial nitric oxide synthase (eNOS), two isoforms of nitric oxide synthetases (NOS) in human placenta, resulting in the increased NO generation. More importantly, TGD administration not only increased the weight gain during pregnancy and revealed a hypotensive effect, but also improved the placental weight gain and attenuated fetal growth restriction in an NG-nitro-L-arginine methyl ester (L-NAME)-induced mouse PE-like model. Our results thereby provide new insights into the role of TGD as a potentially novel treatment for PE.

## Introduction

As a common disorder complicating approximately 4.6% of pregnancies, preeclampsia (PE), also known as EPH-gestosis, is a pregnancy-specific syndrome, which is defined by the onset of hypertension (systolic pressure ≥ 140/diastolic pressure ≥ 90 mmHg) and proteinuria ( ≥ 0.3 g/24 h ) after 20 weeks of gestation in a previously normotensive woman that also may be associated with myriad, other signs and symptoms, and often with subnormal fetal growth (Ramos et al., 2017; Filipek and Jurewicz, 2018). In recent decades, PE has been considered to be one of the leading causes of maternal morbidity as well as mortality and preterm delivery worldwide. So far several medical conditions have been found to be associated with increased PE risk, including chronic hypertension, diabetes mellitus, renal disease, and hypercoagulable states, and obstetrical conditions with increased placental mass, such as hydatidiform mole and multifetal gestation, increase the risk of PE as well (Lisonkova and Joseph, 2013; Dhariwal and Lynde, 2017). However, the etiology, pathogenesis and treatment of PE remain unclear, while delivery of the placenta is the only known therapeutic strategy for this clinical disease (Dymara-Konopka et al., 2018) while delivery of the placenta is the only known cure and that current therapeutic treatments focus on treating the maternal symptoms (Dymara-Konopka et al., 2018).

While the etiology is not fully understood, it is well known that the first step for the development of PE for many women is the impaired placental cytotrophoblast invasion and inadequate maternal spiral artery remodeling which subsequently results in placental ischemia and hypoxia (Li et al., 2020; Wang et al., 2020). Therefore, vascular dysfunction is central to the pathophysiology of PE, and impairments in NO signaling likely play a key role in driving PE progression and severity. Since NO is required throughout gestation including ovulation, implantation, uterine vascular remodeling, peripheral vascular resistance and vasoreactivity (Thanda et al., 1996; Rengarajan et al., 2020), it is natural to think that NO synthesis and NO signaling are critical for maternal vascular adaptation, vascular maintenance during pregnancy, and for placental and embryonic development.

Tianma Gouteng Decoction (TGD) is made up of 11 herbal remedies, including *Uncaria*, *Gastrodia elata*, *Scutellaria baicalensis* Georgi., *Eucommia ulmoides* Oliv., achyranthes root, *Loranthus parasiticus*, abalone shell, Gardenia, *Leonurus japonicus*, caulis polygoni multiflori, and *Poria cocos* (Liu et al., 2015). Chinese pharmacopoeia (2015 edition) reported that TGD has been widely used in recent decades in the clinical treatment of symptoms caused by hypertension (The State Pharmacopoeia Commission of P. R. China, 2015), such as dizziness and headache, and TGD is considered to be an effective therapeutic strategy for essential hypertension with fewer side effects than western medicine, which is used clinically to effectively lower blood pressure (Ho et al., 2005; Liu et al., 2015; Deng et al., 2022). More recently, a report showed that TGD could protect the endothelial function of the superior mesenteric artery and lower the renal protein expression in spontaneously hypertensive rats (Li et al., 2015), and TGD was found to reduce the loss of dopaminergic neurons in rats with Parkinson’s disease, to inhibit the apoptosis of human SH-SY5Y cells and to exhibit additional neuro-protective effects (Liu et al., 2015), and TGD exerted the anti-apoptotic effect *via* osteoprotegerin (OPG) and tumor necrosis factor related apoptosisinducing ligand (TRAIL), thereby providing cardiovascular protection by a hypotensive mechanism in spontaneously hypertensive rats (Dong et al., 2020), indicating the potentially wide use of TGD for the treatment of various diseases. As PE is closely associated with hypertension during pregnancy, we thereby hypothesized that TGD may alleviate PE progression.

Here, we report that TGD treatment effectively down-regulated the H_2_O_2_-induced ROS levels and significantly up-regulated the H_2_O_2_-suppressed NO production through regulation of TNOS activity as well as the expression of nNOS and eNOS in human extravillous trophoblast-derived TEV-1 cells. More importantly, TGD administration not only increased the weight gain during pregnancy and had a hypotensive effect, but also improved the placental weight gain and attenuated fetal growth restriction in an L-NAME-induced mouse PE-like model. Our results thereby provide new insights into the role of TGD as a novel treatment for PE.

## Methods and Materials

### Human Placental Tissue Collection

Human placental tissue samples [normal, *n* = 10, gestational age at delivery (weeks): 39.3 ± 0.7, and PE, *n* = 10, gestational age at delivery (weeks): 38.5 ± 0.9] were obtained between 29 and 40 weeks of gestation from Jan to Dec of 2019 and provided by Tongde Hospital of Zhejiang Province (Hangzhou, China), following a protocol approved by the Ethics Committee of Tongde Hospital of Zhejiang Province. Patients with PE were defined as having systolic and diastolic blood pressure > 140 and 90 mm Hg, respectively, measured at least 6 h apart plus proteinuria ≥ 300 mg/24 h or > 1+ by dipstick test. Clinical states of known pathogenesis which could possibly interfere with values of studied parameters were excluded. Exclusion criteria were also uterine contractions and premature rupture of amniotic membranes. To get similar clinical condition with PE, control group with similar gestational age were collected. Written informed consent was obtained for each participant.

### Determination of Dihydroethidium in Human Placental Samples

Dihydroethidium (DHE, O_2_·) was determined using a DHE assay kit (Beyotime, China) as described previously (Huang et al., 2013). Placental explants were washed with PBS, incubated in fresh culture medium containing 2 μM DHE for 30 min at 37°C and washed three times. Fluorescence intensity was measured with a fluorescence microscope, with the excitation wavelength of 535 nm.

### Preparation of Tianma Gouteng Decoction Extract

Dry materials of the ingredients in proportions as listed here were pulverized. One dose of TGD for human is: Gastrodiae Rhizoma (Tianma) 9 g, Uncaria Ramulus Cum Uncis (Gouteng) 12 g, Haliotidis Concha (Shijueming) 18 g, Gardeniae Fructus (Zhizi) 9 g, Scutellariae Radix (Huangqin) 9 g, Cyathulae Radix (Chuanniuxi) 12 g, Eucommiae Cortex (Duzhong) 9 g, Leonuri Herba (Yimucao) 9 g, Taxilli Herba (Sangjisheng) 9 g, Polygoni Multiflori Caulis (Shouwuteng) 9 g, Poria (Fuling) 9 g (The State Pharmacopoeia Commission of P. R. China, 2015). After pulverizing, the powder was steeped with threefold tap water (v/w) for 0.5 h, boiled for 1 h, and then filtered. The residue was extracted with tap water and the filtrate was concentrated by rotary evaporation under vacuum in a 60°C water bath. The concentrated extract was frozen in liquid nitrogen and finally subjected to lyophilisation under vacuum. Final yield was powdered and then stored at −20°C. All medical materials were purchased from Zhejiang Academy of Traditional Chinese Medicine.

### Cell Lines and Cell Culture

The human first-trimester extravillous trophoblast TEV-1 cell line was obtained from the American Type Culture Collection (ATCC, Manassas, VA) and was incubated in Dulbecco’s 1:1 modified Eagle’s medium/Ham’s F12 culture medium (Life Technologies) supplemented with 10% (v/v) fetal bovine serum (FBS, Life Technologies, Inc., Grand Island, NY) at 37°C in an atmosphere of 5% CO_2_.

### Antibodies, Reagents and shRNA Sequences

Antibodies for nNOS (mouse, sc-5302), iNOS (mouse, sc-7271) and cGMP (mouse, sc-21727) were purchased from Santa Cruz Biotechnology (Santa Cruz, CA), eNOS antibody (rabbit, AF6792) and αTubulin antibody (rabbit, AF0001) were from Beyotime Biotechnology (Shanghai, China), and antibodies for ICAM1 (rabbit, bs-4615R) and NF-κB (mouse, bsm-33059M) were from Bioss (Beijing, China). H_2_O_2_ was obtained from Beyotime Biotechnology (Shanghai, China) and L-NAME was from Selleck (Shanghai, China). ShRNA sequences used are as follows: shRNA-nNOS (CAG​AGA​ATA​GTT​CAC​ATC​TAT), shRNA-iNOS (ATC​GAA​TTT​GTC​AAC​CAA​TAT), shRNA-eNOS (CAG​GAA​GAA​GAC​CTT​TAA​AGA), and a scrambled shRNA sequence (TTT​GTA​CTA​CAC​AAA​AGT​ACT​G) was used as control.

### Standardization of TGD Concentration and Treatment Period

In order to confirm the appropriate concentration and time-course of TGD administration, we treated TEV-1 cells with three different concentrations, including L (25 mg/ml), M (50 mg/ml) and H (100 mg/ml) (Liu et al., 2015), and with different time periods, including 12, 24 and 48 h. Cell Counting Kit-8 (CCK-8) assay was employed to evaluate cell proliferation and the concentration and treatment period of TGD that had no significant effect on TEV-1 cells proliferation was chosen for further experiments.

### CCK-8 Assays

CCK-8 assay was performed according to the manufacturer’s protocol (Yeasen, Shanghai, China) as described previously (Jian et al., 2021). After treatment, TEV-1 cells were incubated with 10 μl CCK-8 reagent for 2 h at 37°C and the absorbance was measured at a wavelength of 450 nm with a micro-plate reader.

### Establishment of Oxidative Stress Models

TEV-1 cells were seeded into the 96-well plate at the density of 5 × 10^3^ cells per well, and cultured at 37°C for 24 h. After the treatment with different concentrations of TGD for 24 h, TEV-1 cells were exposed to different concentrations of H_2_O_2_ (150 μM, 300 μM or 500 μM) for 3 h in the presence or absence of TGD. Six replicated wells were set for each group.

### Intracellular ROS Assays

Intracellular ROS levels were measured by oxidation-sensitive fluorescent probe 2′,7′-dichlorofluorescein-diacetate (DCFH-DA) following the instructions (Beyotime Biotechnology, Shanghai, China). Briefly, TEV-1 cells were cultured in 96-well plates and were collected after treatment. Then, the TEV-1 cells or placental tissue explants were washed with PBS, and were subsequently incubated in 10 μM DCFH-DA for 20 min at 37°C. Fluorescence was quantified by a micro-plate reader with the excitation wavelength of 488 nm and the emission wavelength of 525 nm.

### Nitric Oxide Assays

The levels of intracellular NO were measured using the Nitric Oxide Assay Kit (Applygen Tech., Beijing, China) as previously reported (Ji et al., 2013). Briefly, TEV-1 cells were seeded in 60 mm plates and incubated overnight. Then TEV-1 cells were transfected with control shRNA, nNOS shRNA, iNOS shRNA or eNOS shRNA, and treated with 500 μM H_2_O_2_ for 3 h or with 100 μM L-NAME for 12 h, followed by the incubation with TGD for 24 h. After incubation, Griess reagent was added and the resultant color development was quantified by reading the absorbance at 550 nm. For measurements in placental samples, placental explants were cultured in 12-well plates for 48 h, then the medium was collected and the Griess reagent was added. The resultant color development was quantified by reading the absorbance at 550 nm.

### Measurement of Superoxide Dismutase Activity

The activity for SOD was determined using the xanthine oxidase assay kits (Nanjing Jiancheng Bioengineering Institute, China) as described previously (Mao et al., 20122012). Briefly, when the treatment was done, TEV-1 cells were washed thoroughly, collected, and lysed on ice with an ultrasound disruptor. Thereafter, the lysed cells were centrifuged at 10,000 g for 10 min, and the total protein concentration of the supernatant was tested using a bicinchoninic acid (BCA) kit (Beyotime Biotechnology, Shanghai, China). The xanthine/xanthine oxidase system was used to produce superoxide anion (O^2−^), which react with 2-(4-iodophenyl)-3-(4-nitrophenol-5-phenlyltetrazolium chloride) to generate a red formazan dye, and the absorbance was measured at a wavelength of 450 nm.

### Measurement of Catalase Activity

CAT activity was measured using a kit (Nanjing Jiancheng Bioengineering Institute, Nanjing, China) according to the manufacturer’s instructions. Briefly, when the treatment was done, TEV-1 cells were washed thoroughly, collected, and lysed on ice with an ultrasound disruptor. Thereafter, the lysed cells were centrifuged at 10,000 g for 10 min, and the total protein concentration of the supernatant was tested using a bicinchoninic acid (BCA) kit (Beyotime Biotechnology, Shanghai, China). H_2_O_2_ is a substrate of a variety of enzymes in cells, including CAT. The CAT activity was thereby determined by analyzing the decomposition rate of H_2_O_2_ caused by CAT and is calculated as units per grams of protein (U/g protein).

### Measurement of Malondialdehyde Activity

The MDA activity was measured using a spectrophotometric diagnostic detection kit (Nanjing Jiancheng Biotechnology Institute, Nanjing, China) as described previously (Li et al., 2014). Briefly, when the treatment was done, TEV-1 cells were washed thoroughly, collected, and lysed on ice with an ultrasound disruptor. Thereafter, the lysed cells were centrifuged at 10,000 g for 10 min, and the total protein concentration of the supernatant was tested using a bicinchoninic acid (BCA) kit (Beyotime Biotechnology, Shanghai, China). MDA was allowed to react with thiobarbituric acid that yielded red-coloured products that were spectrophotometrically quantified by measuring the maximum absorption peaks at 532 nm.

### Measurement of Glutathione Peroxidase Activity

Determination of GSH-PX activities was carried out by kits according to the manufacturer’s instructions (Nanjing Jiancheng Biotechnology Institute, Nanjing, China) as described previously (Lin et al., 2015). Briefly, when the treatment was done, TEV-1 cells were washed thoroughly, collected, and lysed on ice with an ultrasound disruptor. Thereafter, the lysed cells were centrifuged at 10,000 g for 10 min, and the total protein concentration of the supernatant was tested using a bicinchoninic acid (BCA) kit (Beyotime Biotechnology, Shanghai, China). The results were calculated in units of enzymatic activity per milligram of protein (U/mg protein).

### TNOS Activity Assay

The activities of TNOS were assayed using a TNOS assay kit (Nanjing Jiancheng Biotechnology Institute, Nanjing, China) as described previously (Qiu et al., 2018), based on NOS catalysis that converts amino acid L-arginine into citrulline and generates NO. Briefly, when the treatment was done, TEV-1 cells were washed thoroughly, collected, and lysed on ice with an ultrasound disruptor. Thereafter, the lysed cells were centrifuged at 10,000 g for 10 min, and the total protein concentration of the supernatant was tested using a bicinchoninic acid (BCA) kit (Beyotime Biotechnology, Shanghai, China).

### RNA Isolation and Quantitative Real-Time PCR

Total RNA was extracted from TEV-1 cells according to the instructions of the Trizol reagent according to the manufacturer’s instructions (Yeasen, Shanghai, China). 5 μg total RNA in a volume of 20 μl was reversely transcribed to generate cDNA using SuperScript III reagent (Takara Biotechnology, Dalian, China). And then 2 μl of cDNA were used for per quantitative real-time PCR amplification, which was performed on an ABI 7500 Real-Time PCR System (Applied Biosystems, Waltham, MA) according to the qRT-PCR SYBR Green method (Yeasen, Shanghai, China) with the following primers: *nNOS*, F-5′-GGAATCCAGGTGGACAGAGA-3′, R-5′- GGG​CAG​AGG​TTT​GTG​TGA​CT-3’; *iNOS*, F-5′-CAGCGGGATGACTTTCCAAG-3′, R-5′- CAG​CGG​GAT​GAC​TTT​CCA​AG-3’; *eNOS*, F-5′-GGACTTCATCAACCAGTAC-3′, R-5′- GAT​GTA​GGT​GAA​CAT​TTC​C-3’; *ICAM1*, F-5′-GGCATTGTTCTCTAATGTCTCCG-3′, R-5′- CCG​CTC​AGA​AGA​ACC​ACC​TTG​G-3’; *sFLT1*, F-5′-ACAATCAGAGGTGAGCACTGCAA-3′, R-5′- TCC​GAG​CCT​GAA​AGT​TAG​CAA-3’; *αTubulin*, F-5′-ATGCCAAGTGACAAGACCATTG-3′, R-5′- ACT​TCT​TGC​CAT​AAT​CAA​CTG​AGA-3’. The CT value was recorded and the relative expression levels of target genes were normalized to *αTubulin* levels, respectively, to the analyzed using the 2^−ΔΔCt^ method. Each experiment was performed in triplicate.

### Western Blot Analysis For Protein Content Examination

Western blots were performed as described previously (Jin et al., 2021). Briefly, TEV-1 cells were harvested and lysed using NP-40 buffer in the presence of protease inhibitors cocktail (Beyotime Biotechnology, Shanghai, China). Protein concentrations were measured by a bicinchoninic acid (BCA) protein assay kit (Beyotime Biotechnology, Shanghai, China). Then protein was subjected to SDS-PAGE Gel (50 μg protein per lane) and was transferred to polyvinylidene difluoride (PVDF) membranes. Membranes were firstly blocked with 5% non-fat milk for 1 h at room temperature followed by incubation with the corresponding primary antibodies overnight at 4°C. Then membranes were incubated with the secondary antibodies linked to horseradish peroxidase for 1 h at room temperature, and signals were subsequently developed using the Enhanced Chemiluminescence System.

### Animals

Adult male and female C57BL/6 mice weighing 18–20 g were obtained from Cyagen (Suzhou, China), and were provided free access to standard mouse chow and tap water and were maintained under controlled conditions of temperature (23°C), humidity (40–60%), and lighting (12 h/12 h light/dark cycle) for 1 week before the experiments. Vaginal smears were obtained daily from female mice until the vaginal cytologic findings were indicative of estrus. The female mice were mated with male mice at a ratio of 2:1 and assessed for vaginal plugs the following morning, which was designated gestational day 0 (GD 0). On GD 0.5, the female mice were assigned to 4 groups (*n* = 8 each). From gestational day 7.5 (GD 7.5) to GD 18.5, L-NAME (60 mg/kg/d; Selleck, Shanghai, China) or normal saline (similar dose) was administered subcutaneously, and from GD 8.5 to GD 18.5, TGD (dissolved in pure water) at 20 g/kg/d (low dose) or 40 g/kg/d (high dose) or pure water was intragastrically administered (Deng et al., 2020).

### Blood Pressure Measurement

Systolic blood pressures (SBPs) were measured before grouping and on GDs 6.5, 12.5 and 18.5 *via* the indirect method of tail cuff occlusion in conscious animals using a CODA monitor system (Kent Scientific, Torrington, CT) as described previously (Singh et al., 2011). Before each measurement was taken, each mouse was exposed to 37°C for 5 min, and measurements were taken three times for averaging purposes.

### Twenty-Four-Hour Urinary Protein Monitoring

Twenty-four-hour urine was collected with metabolic cages on GDs 6.5 and 18.5. The urine outputs were recorded and the concentration of protein was examined by using a bicinchoninic acid (BCA) protein assay kit (Beyotime, China) according to the manufacturer’s instructions and was presented in absorbance units (AU). Briefly, 10 µl of the samples and standards (bovine serum albumin) were used, and afterwards, 200 µl of the working solution (bicinchoninic acid and copper sulphate, 49:1 ratio, respectively) were added. Then, samples were incubated at 37°C for 30 min. After cooling the samples at room temperature, absorbance was measured at 562 nm. Finally, the 24-h urine protein concentration was calculated and was normalized by urine volume.

### Tissue Preparation and Histopathologic Examination

Pregnancy was terminated on GD 18.5 *via* cesarean delivery after pregnant dams were killed by cervical dislocation under isoflurane. The placentas and delivered pups from the horns of the uterus were examined for weight, length and number, and the resorptions were inspected as well. The isolated placentas were washed with an ice-cold 0.9% saline solution, fixed in 4% paraformaldehyde (PFA) and embedded in paraffin. Four-micrometer paraffin sections were prepared, stained with hematoxylin-eosin and observed under a light microscope (Nikon, Tokyo, Japan).

### Statistical Analysis

Data are presented as means ± S.D. and were analyzed by an unpaired two-tailed *t* test, one-way ANOVA and two-way ANOVA (SPSS 13.0J software; SPSS, Inc., Chicago, IL). Statistical significance was assessed at *p* < 0.05 and *p* < 0.01.

## Results

Increase of reactive oxygen species (ROS) and decrease of nitric oxide (NO) in PE placentas.

**FIGURE 1:**
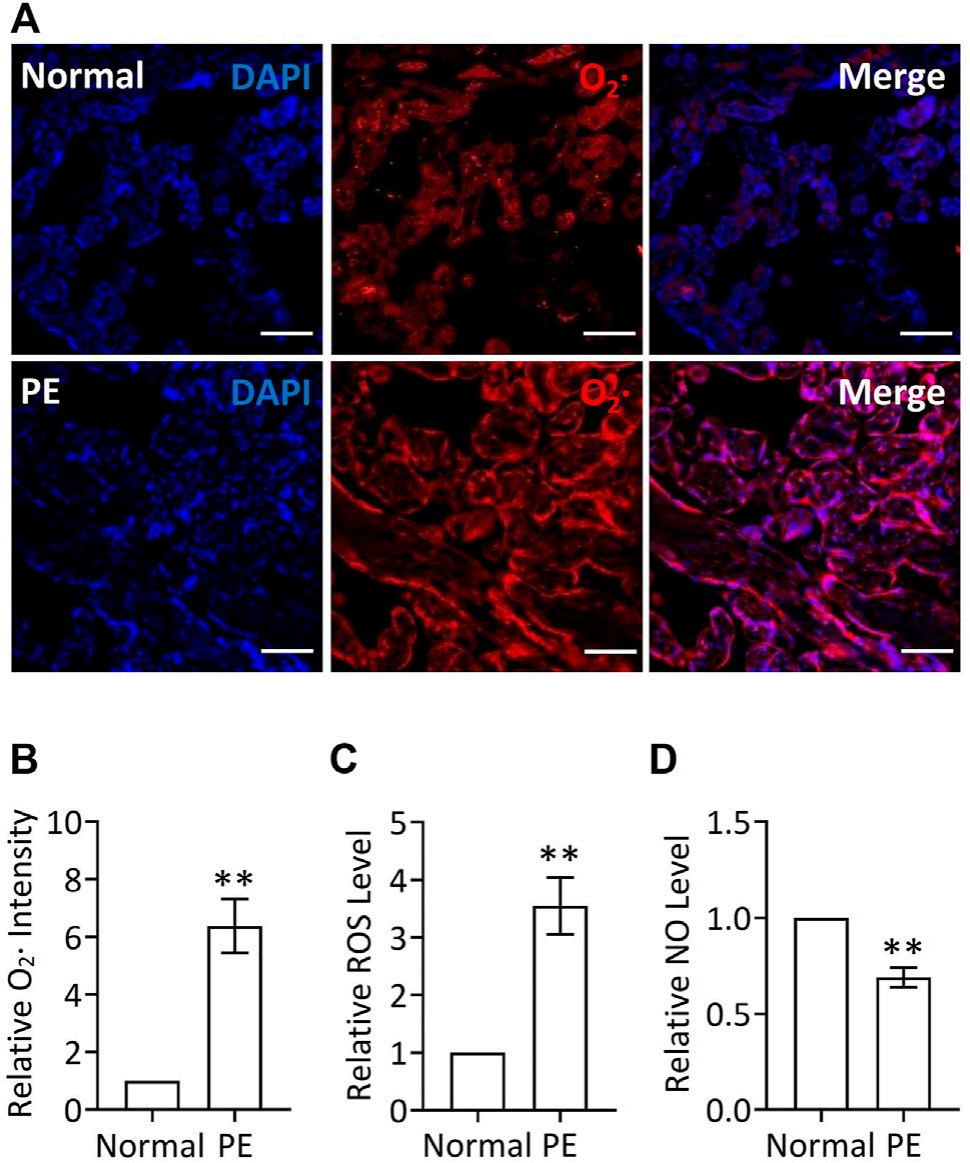
Increased ROS levels and decreased NO levels in PE placenta. **(A)** DHE staining of placental explants from women with normal pregnancy (Normal) or PE. Nuclei were stained with DAPI and red signal indicated O_2_. Bar, 100 μm. **(B)** Quantification *via* staining intensity and statistical analysis of **(A)** (relative to Normal). **(C)** Relative (to Normal) ROS levels in placental explants from women with normal pregnancy (Normal) or PE. **(D)** Relative (to Normal) NO levels in placental explants from women with normal pregnancy (Normal) or PE.

Given that placenta-borne oxidative stress and consequently impaired NO generation are considered as the major molecular determinants of the maternal disease (Aouache et al., 2018), we first examined the superoxide (O_2_·, an important source of ROS) levels using a DHE probe as well as the total ROS production *via* a ROS detection kit in human preeclamptic placental explants. Our results revealed that the ROS production, including O_2_· levels, was significantly increased in preeclamptic explants, compared with that in placental explants from normal pregnancy (Figures 1A–C), indicating the occurrence of oxidative stress in PE, which was consistent with a previous report (Shaheen et al., 2020), whereas NO levels were significantly decreased in preeclamptic placental explants, which was verified by a nitric oxide assay kit (Figure 1D). Thus, it is likely that restoration of ROS production and promotion of NO levels in placenta could be a potentially therapeutic strategy for PE.

### TGD Prevents ROS Production and Elevates NO Levels

**FIGURE 2:**
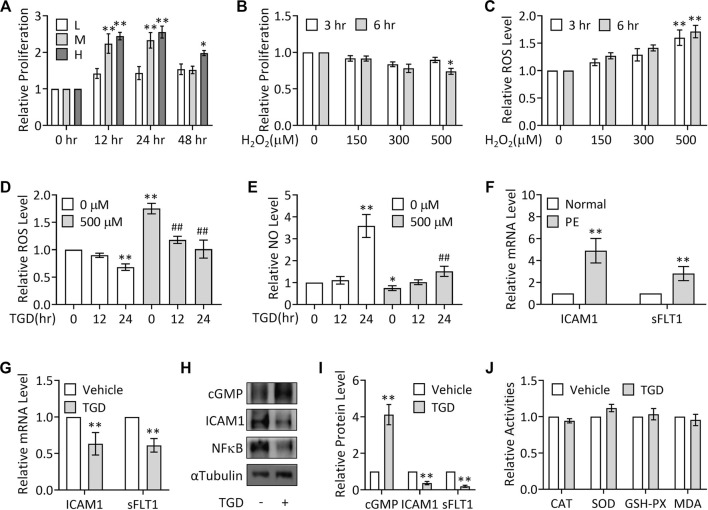
TGD inhibits ROS generation and elevates NO production. **(A)** CCK-8 assays in TEV-1 cells treated with TGD at 25 mg/ml (L), 50 mg/ml (M) or 100 mg/ml (H) for 12, 24 or 48 h **(B)** CCK-8 assays in TEV-1 cells treated with H_2_O_2_ at 150 μM, 300 μM or 500 μM for 3 h or 6 h **(C)** Relative ROS levels in TEV-1 cells treated with H_2_O_2_ at 150 μM, 300 μM or 500 μM for 3 h or 6 h **(D)** Relative ROS levels in TEV-1 cells in the presence or absence of 500 μM H_2_O_2_ for 3 h and then treated with TGD at 25 mg/ml for 12 h or 24 h **(E)** Relative NO levels in TEV-1 cells in the presence or absence of 500 μM H_2_O_2_ for 3 h and then treated with TGD at 25 mg/ml for 12 h or 24 h **(F)** Relative (to Normal) mRNA levels of ICAM1 and sFLT1 in placental explants from women with normal pregnancy (Normal) or PE. **(G)** Relative mRNA levels of ICAM1 and sFLT1 in TEV-1 cells treated with or without TGD at 25 mg/ml for 24 h **(H)** Western blot analysis for cGMP, ICAM1 and sFLT1 in TEV-1 cells treated with or without TGD at 25 mg/ml for 24 h **(I)** Quantification *via* densitometry (*n* = 3) and statistical analysis of bands of **(H)**. **(J)** Relative activities of CAT, SOD, GSH-PX and MDA in TEV-1 cells treated with or without TGD at 25 mg/ml for 24 h.

To investigate the role of TGD in PE treatment, we cultured TEV-1 cells, which were derived from the first-trimester normal extravillous trophoblast cells of a healthy woman placenta, and treated with TGD. In order to determine the appropriate concentration and treatment time for further experiments, we performed CCK-8 assays to first confirm the effect of TGD on TEV-1 cells growth. The results showed that treatment with TGD of either medium dose (M, 50 mg/ml) or high dose (H, 100 mg/ml) significantly promoted TEV-1 cells proliferation at both 12 and 24 h, and high dose TGD-treatment (H, 100 mg/ml) also caused a significant increase in cell proliferation at 48 h, whereas the low dose groups (L, 25 mg/ml) at 12, 24 or 48 h did not present obvious alterations in TEV-1 cell proliferation (Figure 2A). Thus, we chose TGD treatment of low dose (25 mg/ml) for 12 h or 24 h for further experiments.

To next investigate the potential effect of TGD on oxidative stress, H_2_O_2_ was utilized to stimulate ROS generation in TEV-1 cells to mimic the oxidative stress in PE trophoblasts, as superoxide anion generated endogenously or exogenously can rapidly be converted to hydrogen peroxide such as H_2_O_2_ (Veal et al., 2007), and previous studies showed a significant elevation of H_2_O_2_ levels in the bloodstream as well as the placentas of women with PE (Emberton et al., 2003; Steiner and Raghow, 2003; Zhou et al., 2012; Cross et al., 2015). Results from CCK-8 assays revealed that only H_2_O_2_ treatment at 500 μM for 6 h significantly impeded TEV-1 cells proliferation (Figure 2B), while H_2_O_2_ treatment at 500 μM for both 3 and 6 h significantly caused ROS production (Figure 2C). Thus, H_2_O_2_ treatment at 500 μM at 3 h was chosen for further investigations.

Although the H_2_O_2_ treated (500 μM, 3 h) group showed significantly increased ROS production and decreased NO levels (Figures 2D,E), treatment with TGD for 24 h significantly suppressed the ROS levels in the presence or absence of H_2_O_2_ stimulation (Figure 2D), while TGD treatment for 24 h not only significantly elevated NO production but also effectively restored the H_2_O_2_-inhibited NO levels (Figure 2E), suggesting the role of TGD in relief from oxidative and nitrosative stresses in trophoblasts. Consistently, in PE placental tissues, the mRNA expression of intercellular adhesion molecules, such as intercellular adhesion molecule 1 (ICAM1) (Marzolla et al., 2017), and anti-angiogenic factors, such as soluble Fms-Like Tyrosine kinase-1 (sFLT1) (Maynard et al., 2003), was significantly up-regulated (Figure 2F), whereas TGD treatment not only significantly enhanced the content levels of cGMP, the second messenger cyclic guanosine-3′,5′-monophosphate (cGMP) and a key downstream effector of NO (Kastrati et al., 2010), but also significantly suppressed the mRNA and protein expression of ICAM1, decreased sFLT1 mRNA expression and inhibited the protein levels of oxidative stresses-regulated transcription factors such as NFκB (Figures 2G–I), indicating that TGD exerts antioxidant and anti-inflammatory effects.

Given that oxidative stress can also result from a lack of antioxidants, including the enzymatic antioxidants, the major category of antioxidants, such as catalase (CAT), SOD and GSH-PX (Espinosa-Diez et al., 2015), we further examined the effect of TGD on the activities of these antioxidants. Unexpectedly, the activities of CAT, SOD or GSH-PX showed no change in response to TGD treatment, nor did the MDA activity, a molecule involved in oxidative stress pathway (Figure 2J) (Khoubnasabjafari et al., 2015). Thus, TGD prevents ROS generation and promotes NO production.

### TGD Increases NO Levels by Up-Regulation of NOS Activity and NOS Expression

**FIGURE 3:**
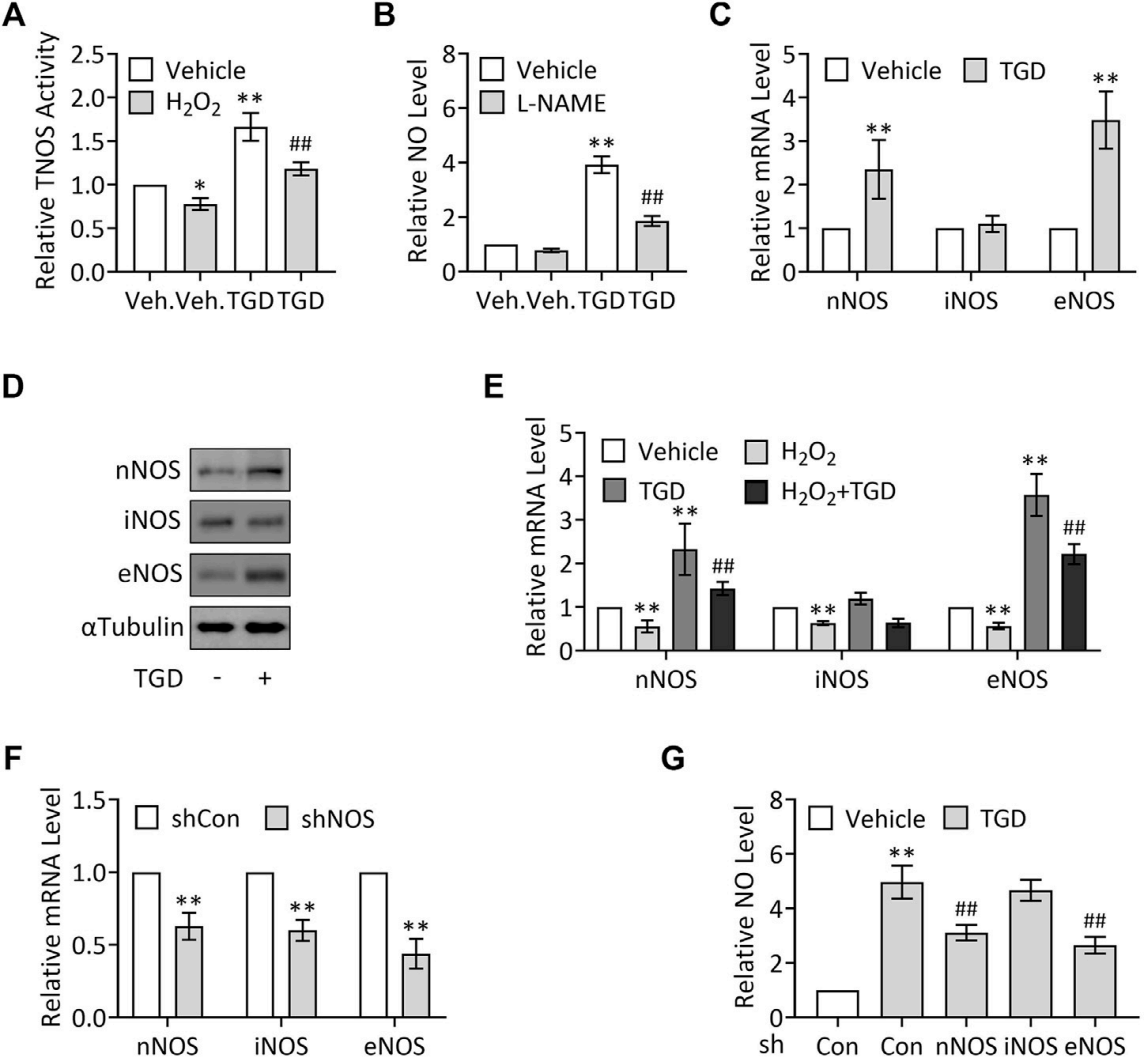
TGD enhances NOS activity and induces the expression of nNOS and eNOS. **(A)** Relative TNOS activity in TEV-1 cells in the presence or absence of 500 μM H_2_O_2_ for 3 h and then treated with or without TGD at 25 mg/ml for 24 h. **(B)** Relative NO levels in TEV-1 cells in the presence or absence of 100 μM L-NAME for 12 h and then treated with or without TGD at 25 mg/ml for 24 h **(C)** Relative mRNA levels of nNOS, iNOS and eNOS in TEV-1 cells treated with or without TGD at 25 mg/ml for 24 h **(D)** Western blot analysis for nNOS, iNOS and eNOS in TEV-1 cells treated with or without TGD at 25 mg/ml for 24 h **(E)** Relative mRNA levels of nNOS, iNOS and eNOS in TEV-1 cells in the presence or absence of 500 μM H_2_O_2_ for 3 h and then treated with or without TGD at 25 mg/ml for 24 h **(F)** Relative mRNA levels of nNOS, iNOS and eNOS in TEV-1 cells transfected with control shRNA, nNOS-shRNA, iNOS-shRNA or eNOS-shRNA and cultured for 72 h **(G)** Relative NO levels in TEV-1 cells transfected with nNOS-shRNA, iNOS-shRNA or eNOS-shRNA and cultured for 48 h, followed by treatment with or without TGD at 25 mg/ml for 24 h.

NO is synthesized from L-arginine under the action of NOS, which catalyze L-arginine oxidation into NO and L-citrulline (Knowles and Moncada, 1994; Förstermann and Sessa, 2012). To explore the mechanisms underlying TGD in regulation of NO production, we examined the effect of TGD on TNOS enzymatic activity using a commercial kit. Our results exhibited that H_2_O_2_ treatment (500 μM, 3 h) significantly suppressed TNOS activity, which was consistent with a previous report (Kotsonis et al., 1999), while TGD treatment not only significantly up-regulated TNOS activity but also effectively restored the H_2_O_2_-suppressed TNOS activity (Figure 3A), indicating TGD regulates TNOS activity. Consistently, treatment with L-NAME, an inhibitor of TNOS activity (Pfeiffer et al., 1996), significantly lowered the TGD-induced NO levels, compared with the TGD-treatment group (Figure 3B). Thus, TGD promotes NO generation by up-regulation of TNOS activity.

Given that there are at least three NOS proteins encoded by distinct genes in human placenta, including nNOS, immunological (inducible) nitric oxide synthase (iNOS), and eNOS (Di Iulio et al., 1999; Yang et al., 2005), we next want to know whether all the three NOS isoforms are involved in TGD-regulated NO generation. To this end, we first examined the corresponding expression of the three NOS in response to TGD treatment, respectively. Unexpectedly but intriguingly, TGD significantly up-regulated the mRNA and protein levels of nNOS and eNOS, while iNOS expression remained unchanged in the presence of TGD (Figures 3C,D). Moreover, H_2_O_2_ treatment (500 μM, 3 h) suppressed the mRNA expression of all the three NOS isoforms, among which, the expression of nNOS and eNOS could be effectively attenuated by TGD treatment for 24 h (Figure 3E), indicating nNOS and eNOS may participate in TGD-mediated NO production. On the other hand, we constructed plasmids expressing NOS-shRNAs which knocked down the mRNA expression of nNOS, iNOS or eNOS by as much as 40–60% (Figure 3F), and observed the alterations in NO production in NOS shRNAs-transfected TEV-1 cells, respectively. Consistently, knockdown of nNOS or eNOS but not iNOS significantly down-regulated the TGD-induced NO levels (Figure 3G). Thus, TGD promotes NO generation by up-regulation of the expression of nNOS and eNOS.

### Relief of Hypertension and Decrease of Urinary Protein Concentration in a PE Mouse Model by TGD

**FIGURE 4:**
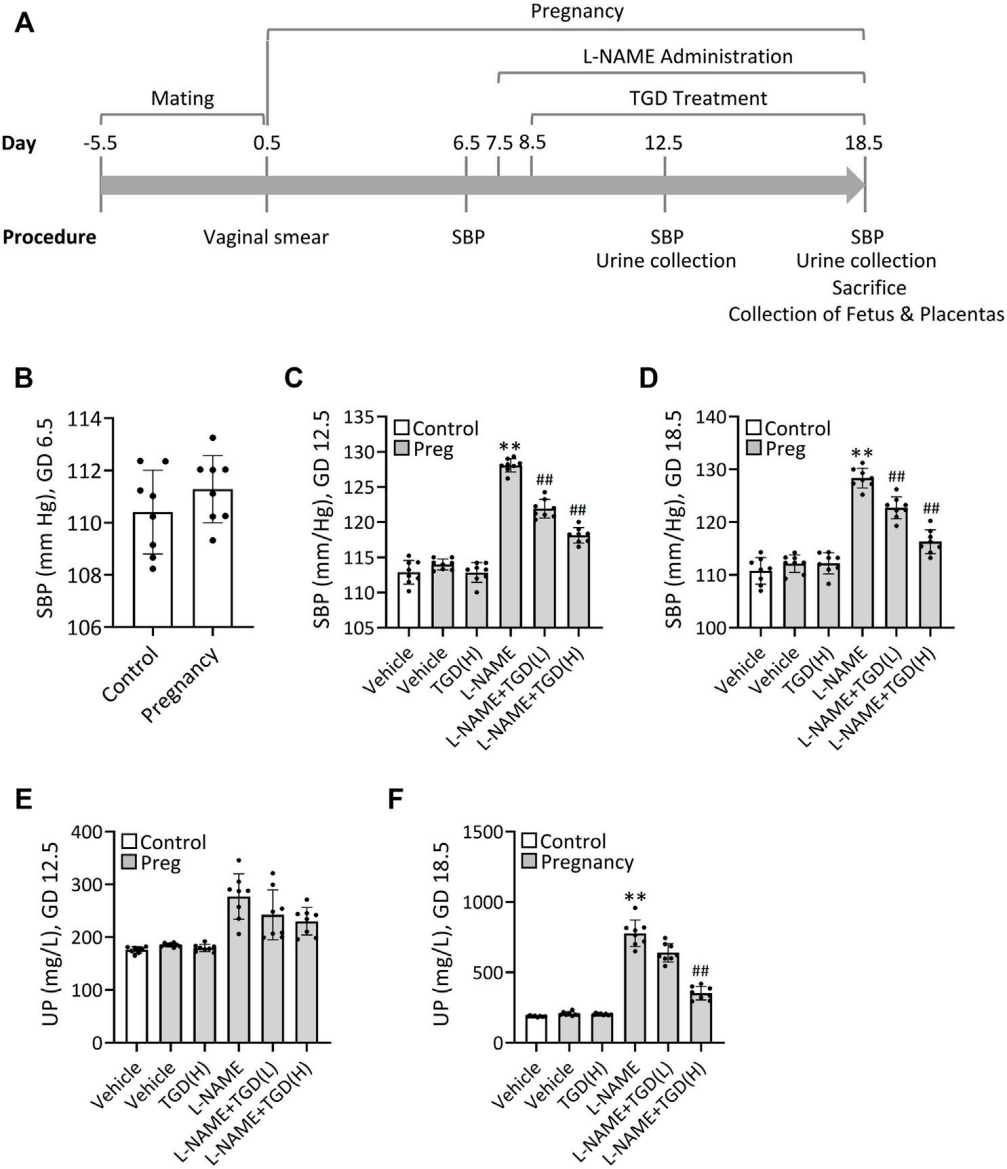
TGD relieves hypertension and decreases urinary protein concentration in a PE mouse model. **(A)** Schematization of the procedures and treatments included in the protocol over time. SBP, systolic blood pressure. **(B)** SBP values in unpregnant (control) and pregnant mice (Pregnancy) at gestational day (GD) 6.5. **(C)** SBP values in unpregnant (control) and pregnant mice (Preg) administrated with or without L-NAME and TGD at low dose (L, 20 g/kg/d) or high dose (H, 40 g/kg/d) at GD 12.5. **(D)** SBP values in unpregnant (control) and pregnant mice (Preg) administrated with or without L-NAME and TGD at low dose (L, 20 g/kg/d) or high dose (H, 40 g/kg/d) at GD 18.5. **(E)** Urinary protein concentrations (UP) in unpregnant (control) and pregnant mice (Preg) administrated with or without L-NAME and TGD at low dose (L, 20 g/kg/d) or high dose (H, 40 g/kg/d) at GD 12.5. **(F)** Urinary protein concentrations (UP) in unpregnant (control) and pregnant mice (Pregnancy) administrated with or without L-NAME and TGD at low dose (L, 20 g/kg/d) or high dose (H, 40 g/kg/d) at GD 18.5.

To verify the therapeutic effect of TGD on PE *in vivo*, we next took advantage of the L-NAME-induced PE mouse model as described previously (Zhao et al., 2018). Schematization of the procedures and treatments was shown in Figure 4A. Results revealed that the mean systolic blood pressure (SBP) on GD 6.5 were 110 ± 1.6 mm Hg in the control group without pregnant and 112 ± 1.6 mm Hg in the group of pregnant mice awaiting L-NAME treatment (Figure 4B). Once L-NAME was administered, the mean SBP of the pregnant mice in the L-NAME group was increased significantly on GD 12.5 and maintained until GD 18.5, compared with the control group (Pregnancy, vehicle) (Figures 4C,D), while administration of TGD at both low dose and high dose significantly lowered the SBP on GD 12.5 and GD 18.5 , compared with the L-NAME group (Figures 4C,D), suggesting the hypotensive effect of TGD during pregnancy. Although the 24-h urinary protein concentration did not show significant change between the control group (Pregnancy, vehicle) and the L-NAME group on GD 12.5 (Figure 4E), administration of L-NAME significantly increased the 24-h urinary protein concentration on GD 18.5 (Figure 4F), which was effectively restored by administration of TGD at a high dose (Figure 4E). Of note, neither the mean SBP nor the 24-h urinary protein concentration in the control group (Pregnancy, vehicle) was significantly altered by TGD administration (Figures 4C–F).

### Restoration of Pregnancy Outcome in a PE Mouse Model by TGD

**FIGURE 5:**
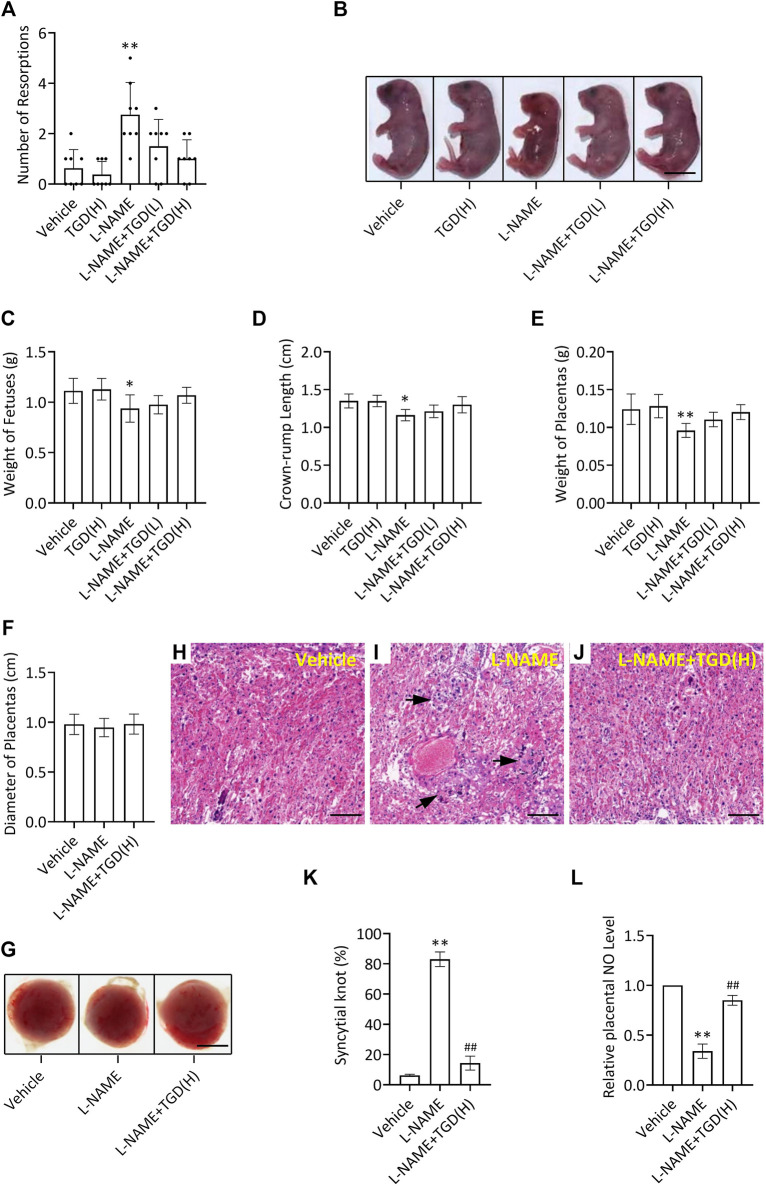
TGD restores pregnancy outcome in a PE mouse model. **(A)** Number of resorptions pregnant mice administrated with or without L-NAME and TGD at low dose (L, 20 g/kg/d) or high dose (H, 40 g/kg/d). **(B)** Representative pictures of fetuses from pregnant mice administrated with or without L-NAME and TGD at low dose (L, 20 g/kg/d) or high dose (H, 40 g/kg/d). Bar, 0.6 cm. **(C)** Weight of fetuses from pregnant mice administrated with or without L-NAME and TGD at low dose (L, 20 g/kg/d) or high dose (H, 40 g/kg/d). **(D)** Crown-rump length of fetuses from pregnant mice administrated with or without L-NAME and TGD at low dose (L, 20 g/kg/d) or high dose (H, 40 g/kg/d). **(E)** Weight of placentas from pregnant mice administrated with or without L-NAME and TGD at low dose (L, 20 g/kg/d) or high dose (H, 40 g/kg/d). **(F)** Diameter of placentas from pregnant mice administrated with or without L-NAME and TGD at high dose (H, 40 g/kg/d). **(G)** Pictures of placentas from pregnant mice administrated with or without L-NAME and TGD at high dose (H, 40 g/kg/d). Bar, 0.25 cm. **(H–J)** H&E staining for placental tissue sections from pregnant mice administrated with or without L-NAME and TGD at high dose (H, 40 g/kg/d). Bar, 50 μm. **(K)** Syncytial knot percentage (%) in placentas from pregnant mice administrated with or without L-NAME and TGD at high dose (H, 40 g/kg/d). **(L)** Relative NO levels in placentas from pregnant mice administrated with or without L-NAME and TGD at high dose (H, 40 g/kg/d). **, ^##^ *p* < 0.01; error bar, SD; *n* = 30.

Compared with the control group (vehicle), the number of resorptions in the L-NAME group was increased, which was down-regulated by TGD administration at either a low dosage or a high dosage (Figure 5A), while no malformations were observed in the fetuses in all groups (Figure 5B). In addition, the mean weight of the fetuses, the crown-rump length of the fetuses and weight of the placentas in the L-NAME group were significantly decreased compared with the control group (vehicle), which were partially up-regulated by TGD administration of both dosages (Figures 5C–F). Although the diameters of the placentas did not present significant differences (Figure 5G), morphologic changes in the placenta could be observed under a light microscope (Figures 5H–J). Placental villi formed a complicated labyrinth-like structure with maternal blood cells, which occupied the spaces of the mesh and contributed to the exchange of the material and nutrition between the maternal and fetal blood. On GD 18.5, normal histologic structures were observed in the control group (vehicle), whereas increased syncytial knots were noted in the L-NAME administration group (Figures 5H–K), resulting in the consequently decreased blood flow in the placenta. However, noticeably, the number of syncytial knots was reduced when TGD administration particularly at the high dosage was applied in the L-NAME group, compared with L-NAME administration alone (Figures 5H–K). Additionally, we also examined the NO levels in mouse placentas and our results exhibited that, although L-NAME significantly suppressed NO levels, TGD administration effectively retrieved that to a comparable level to the vehicle control group (Figure 5L), which was in accordance with the *in vitro* data (Figure 3B). In summary, TGD restores the pregnancy outcome in a PE mouse model.

## Discussion

Since many lines of evidence suggest that NO deficiency contributes to the pathophysiology of PE, it is logic and has been hypothesized that agents that increase NO directly or preserve NO bioavailability may prevent PE. However, NO donor glyceryl trinitrate has been reported to be ineffective and underpowered for preventing PE (Lees et al., 1998; Davis and Brown, 2001). In addition, arginine supplementation has been found to decrease blood pressure, but also markedly contributes to intestinal distress (Gui et al., 2014). Currently, elevations in ROS and oxidative stress have been found in PE, which may lead to impaired NO activity. However, although smaller trails of anti-oxidants showed a potential benefit, larger randomized clinical trials have not demonstrated a benefit in reducing the incidence of PE. Further work is needed to understand the pathophysiology about NO signaling in PE.

Tianma Gouteng Yin (TGY) is one of the most famous traditional Chinese medicines (TCM), which is traditionally used in the form of decoction (TGD) and is commonly prescribed by TCM practitioners to treat hypertension and Parkinson’s disease (PD)-like symptoms such as tremor and paralysis (Liu et al., 2015). Considering PE is a human-pregnancy-specific disease with the occurrence of hypertension, in this study we investigated the potential therapeutic effect of TGD on PE by using a PE-like mouse model and the underlying pregnancy-protective mechanism was also analyzed *in vitro* using human trophoblast-derived TEV-1 cells, providing with the experimental evidences supporting the application of TGD for the treatment of PE.

Previous studies have indicated the role of NOS-NO signaling in PE with controversial results. We hypothesize that the discrepancies in the levels of NOS and NO in PE are likely explained by several factors, such as dietary intake, renal clearance and iron status. As NO is highly sensitive to variations in diet, and specific to the pathophysiology of PE, measurement of circulating NO is only reflective of its production in the steady state (Sutton et al., 2020). Oxidative stress is observed in PE, where the levels of ROS are elevated, resulting in impaired NO signaling and vascular dysfunction. In PE, excess levels of all ROS are reported to be more prevalent, including superoxide superoxide (O_2_·), hydrogen peroxide (H_2_O_2_), hydroxyl radical (OH) and peroxynitrite (ONOO−) (Agarwal et al., 2005; Rozas-Villanueva et al., 2020). In our work presented, we successfully constructed an oxidative stress model *in vitro* in cultured TEV-1 cells by H_2_O_2_ treatment (500 μM, 3 h) that significantly induced ROS levels to 1.8 folds, which is consistent with previous reports (Haendeler et al., 2005; Gao et al., 2020).

NO is synthesized from the amino acid L-arginine by NOS. Three NOS isoforms have been discovered, including eNOS, nNOS and iNOS, among which eNOS is largely stimulated by some other factors, such as platelet derived factors, shear stress, estrogen, growth factors and cytokines, and nNOS and iNOS are involved in the regulation of cell signaling pathways related to vascular homeostasis during pregnancy (Wang et al., 2010; Förstermann and Sessa, 2012). A previous study showed that considerable amounts of NOS activity were detected in both human trophoblastic villi and extravillous cells, suggesting a physiologic role of trophoblastic NO in early stages of pregnancy (Al-Hijji et al., 2003). In addition, eNOS and iNOS have been found in diverse species since early stages of placental development, and eNOS is also expressed in syncytiotrophoblasts in human first trimester placenta (Krause et al., 2011). Another study from Skarzinski *et al.* demonstrated that iNOS was prominently expressed in trophoblasts of the placenta, while nNOS expression was found in scattered trophoblastic cells (Skarzinski et al., 2009). Thus, the three isoforms of NOS, including iNOS, nNOS and eNOS, are all produced by first trimester trophoblast cells. In addition, we discovered that the expression of all the three NOS isoforms, including iNOS, was suppressed by hydrogen peroxide incubation in TEV-1 cells, which is not consistent with a previous report showing that the serum iNOS levels were increased in PE patients (Kim et al., 2020). We hypothesis that the inconsistence could be due to the complicated cellular pathways functioning during PE progression in patients, since iNOS expression is also regulated by other molecules such as Tumor Necrosis Factor-α (TNF-α) that is stimulated in PE (Kim et al., 2020).

It has been suggested that NO serves as the main vasodilatory agent in the placenta during pregnancy that mediates placental development by contributing to cytotrophoblast invasion, implantation, adhesion, aggregation of intervillous platelets as well as placental perfusion (Zullino et al., 2018). Given that NO is mainly produced by extravillous trophoblasts as they invade spiral arteries in the maternal uterus to create an efficient, low-resistance placental unit for adequate feto-placental blood flow (Ariel et al., 1998), in our study present here we examined the effect of TGD on NO production by use of TEV-1 cell line that is derived from human extravillous trophoblasts. Our results revealed that TGD treatment significantly promoted NO production at different periods by up-regulation of the TNOS activity as well as the expression of nNOS and eNOS but not iNOS, suggesting the potential role of TGD in NOS-associated factors and/or cytokines regulation during pregnancy, which merits further investigations.

To validate the data from *in vitro* experiments, we also successfully generated a C57BL/6 background mouse model that simulated the clinical manifestations of PE (hypertension and proteinuria) using the NO inhibitor L-NAME at doses of 60 mg/kg/day that was administered subcutaneously from gestational day 7.5–18.5 of pregnancy. In this model, we evaluated *in vivo* if the effect of administration of TGD could lessen the clinical manifestations of PE. The role of TGD in PE development is not clear yet and there are no studies investigating the effect of TGD administration during human pregnancy or in an animal model of PE. However, the hypotensive and pregnancy-protective effect of TGD administration observed in our study is in agreement with the previous data showing that TGD administration in a hypertension mouse model induces a significant reduction in BP (Dong et al., 2020). In addition, we further explored the possible molecular mechanisms and discovered that the hypotensive effect of TGD is attributed at least partly by the promotion of release of NO in trophoblasts. On the other hand, our data also implied that TGD administration contributed to the pregnancy maintenance in PE condition, with the increased number of fetuses as well as the comparable size and weight of the fetuses to normal group, suggesting the potential application value in clinic of TGD in PE treatment, although TGD administration did not show obvious effect in normal pregnancy mice. Further clinical studies are in need to confirm the TGD treatment in PE patients. Although TGD is able to induce NO through regulation of NOS and L-NAME inhibits NOS activity, our data *in vitro* have revealed that, in the presence of L-NAME, TGD-induced NO levels are still higher than the vehicle control group (without TGD and without L-NAME). We guess that L-NAME only diminished the activity from a part of NO, while the NO amount that TGD produced was far more than L-NAME at that dose can effectively suppress. On the other hand, the protective-effect of TGD against PE is not limited to its regulation of NO generation, TGD also effectively down-regulated the ROS levels, which contributes to the restoration from PE as well.

In summary, in the present study, we demonstrated that administration of TGD in a mouse PE-like model increased the weight gain during pregnancy and had a hypotensive effect, improved the placental weight gain and attenuated fetal growth restriction. Moreover, we also clarified that these effects by TGD are possibly through regulation of TNOS activity as well as the expression of nNOS and eNOS that controls NO production in placental trophoblasts. Thus, these results provide new insights into the role of TGD as a novel treatment for PE.

## Data Availability

The original contributions presented in the study are included in the article/Supplementary Material, further inquiries can be directed to the corresponding author.
