# Supplementary figures and images for: Tianma Gouteng Decoction Exerts Pregnancy-Protective Effects Against Preeclampsia via Regulation of Oxidative Stress and NO Signaling
Source: Front Pharmacol. 2022 Mar 21;13:849074. doi: 10.3389/fphar.2022.849074 (PMC8985411; doi:10.3389/fphar.2022.849074)

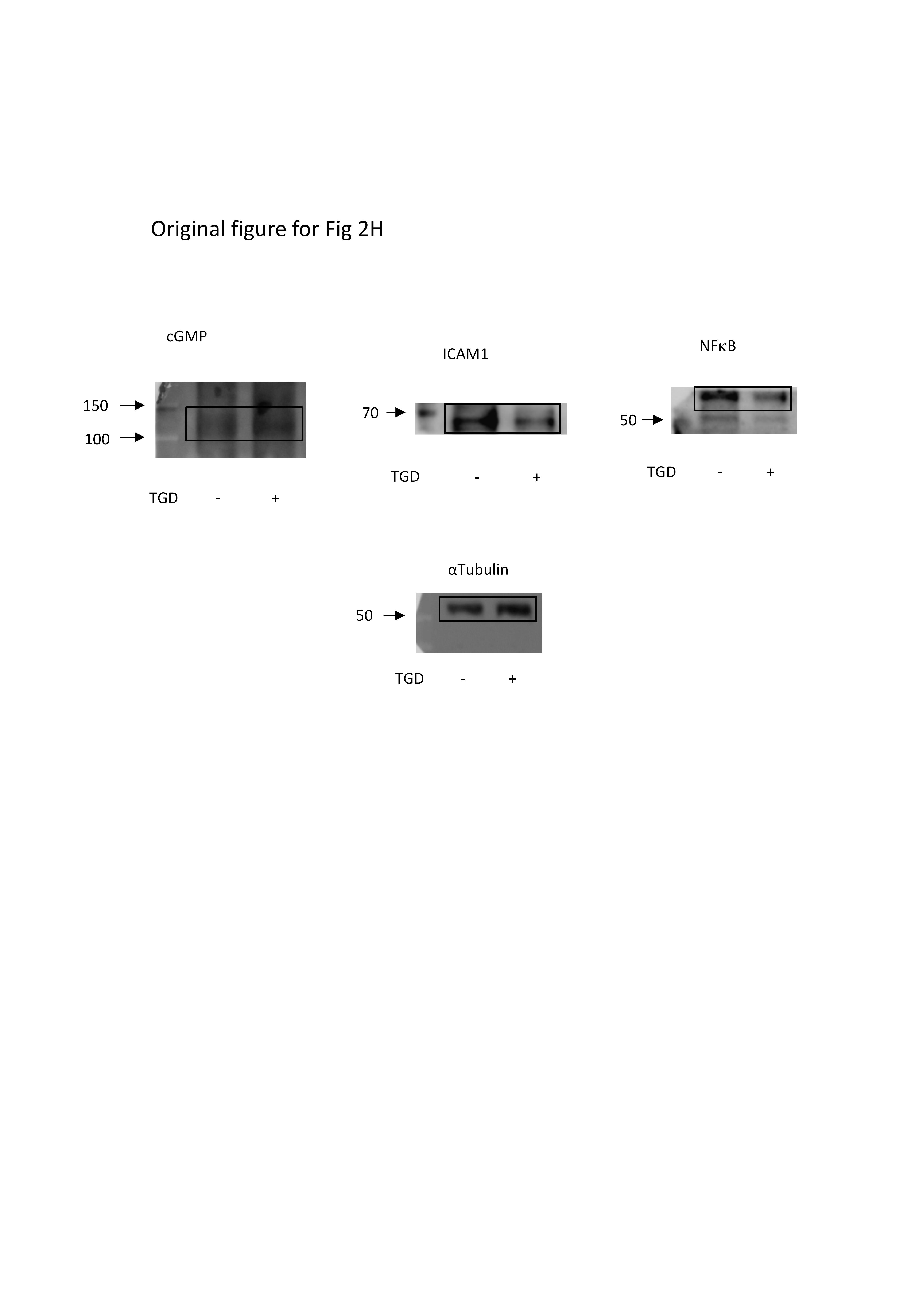

Supplement: Supplementary file 1 [file Image1.JPEG]
